# Mechanisms and Severity of Exercise Intolerance Following COVID-19 and Similar Viral Infections: A Comparative Review

**DOI:** 10.7759/cureus.39722

**Published:** 2023-05-30

**Authors:** Edward J Weldon, Bradon Hong, Jeffrey Hayashi, Connor Goo, Enrique Carrazana, Jason Viereck, Kore Liow

**Affiliations:** 1 Department of Neurology, University of Hawaii John A. Burns School of Medicine, Honolulu, USA; 2 Brain Research, Innovation, & Translation Laboratory, Hawaii Pacific Neuroscience, Honolulu, USA; 3 Department of Neurology, Hawaii Neuro COVID Clinic, Honolulu, USA

**Keywords:** bed rest, deconditioning, viral febrile illness, exercise intolerance, post-acute covid-19 syndrome (pacs)

## Abstract

Approximately 19% of the population is suffering from “Long COVID”, also known as post-acute sequelae of severe acute respiratory syndrome coronavirus 2 (SARS-CoV-2) (PASC), which often results in exercise intolerance. As COVID infections continue to be common, studying the long-term consequences of coronavirus disease (COVID) on physical function has become increasingly important. This narrative review will aim to summarize the current literature surrounding exercise intolerance following COVID infection in terms of mechanism, current management approaches, and comparison with similar conditions and will aim to define limitations in the current literature.

Multiple organ systems have been implicated in the onset of long-lasting exercise intolerance post-COVID, including cardiac impairment, endothelial dysfunction, decreased VO_2 max_ and oxygen extraction, deconditioning due to bed rest, and fatigue. Treatment modalities for severe COVID have also been shown to cause myopathy and/or worsen deconditioning. Besides COVID-specific pathophysiology, general febrile illness as commonly experienced during infection will cause hypermetabolic muscle catabolism, impaired cooling, and dehydration, which acutely cause exercise intolerance. The mechanisms of exercise intolerance seen with PASC also appear similar to post-infectious fatigue syndrome and infectious mononucleosis. However, the severity and duration of the exercise intolerance seen with PASC is greater than that of any of the isolated mechanisms described above and thus is likely a combination of the proposed mechanisms. Physicians should consider post-infectious fatigue syndrome (PIFS), especially if fatigue persists after six months following COVID recovery. It is important for physicians, patients, and social systems to anticipate exercise intolerance lasting for weeks to months in patients with long COVID. These findings underscore the importance of long-term management of patients with COVID and the need for ongoing research to identify effective treatments for exercise intolerance in this population. By recognizing and addressing exercise intolerance in patients with long COVID, clinicians can provide proper supportive interventions, such as exercise programs, physical therapy, and mental health counseling, to improve patient outcomes.

## Introduction and background

Coronavirus disease 2019 (COVID-19) is on track to be the third leading cause of death in the United States for the third year in a row [1]. The impact of COVID is undeniable, but infection rates and mortality do not show a complete picture. Up to 19% of COVID survivors are suffering from "Long COVID", also known as post-acute sequelae of severe acute respiratory syndrome coronavirus 2 (SARS-CoV-2) (PASC), broadly characterized by any symptoms (e.g. dyspnea, headache, cognitive changes) not present before infection and lasting three or more months, as defined by the Centers for Disease Control and Prevention (CDC) [2]. Like COVID, PASC can involve a myriad of systems, commonly affecting the brain, heart, lungs, kidneys, and muscular system [3].

The most common symptoms of long COVID are fatigue and shortness of breath, regardless of the length of infection, disease severity, or time since disease onset, and many patients experience exercise intolerance following COVID infection and recovery. Roughly 90% and 95% of COVID-19 survivors experience symptoms of shortness of breath and fatigue, respectively [4]. As COVID infections continue to be common, studying the long-term consequences of COVID on physical function has become increasingly important.

Exercise intolerance is a complex clinical symptom that is defined as an inability to perform exercise due to decreased ability of the cardiovascular system to supply oxygen to the body, a decreased ability of the musculoskeletal system to effectively use the delivered oxygen during exercise, or both, resulting in decreased oxygen consumption during physical activity. This review will aim to summarize the current literature surrounding exercise intolerance following COVID infection in terms of mechanism, current management approaches, and comparison to similar conditions, and will aim to define limitations in the current literature.

## Review

A narrative review of the literature was conducted by searching electronic databases including PubMed and Google Scholar. The search was conducted using relevant keywords and phrases, such as “exercise intolerance” with “COVID”, “Post-Infectious Fatigue Syndrome”, “EBV”, “Influenza”, or “Febrile Illness”. Relevant articles were critically evaluated for their relevance and quality, and key findings were synthesized into a coherent narrative to compare the mechanism and severity of exercise intolerance seen post-COVID and other infectious illnesses.

Mechanisms of exercise intolerance due to COVID infection

Inflammation in the respiratory tract due to COVID infection can lead to pulmonary edema and diffuse alveolar lesions. Prolonged use of a ventilator can also contribute to pulmonary injury, causing edema, impaired surfactant function, and decreased lung compliance, leading to decreased respiratory function [5]. Decreased lung function is exacerbated by the consolidation of lung parenchyma due to healing and fibrosis following infection. Together, these factors appear to contribute to the shortness of breath and fatigue seen following COVID infection [4].

Although COVID is primarily a disease of the respiratory tract, recent evidence has shown that COVID infection can affect the entire cardiovascular and endothelial system. It has been well-established that COVID can infect the angiotensin-converting-enzyme 2 endothelial receptors in the heart, lungs, and vasculature. COVID infiltrates cells mainly through the angiotensin-converting-enzyme 2 receptor, which is a ubiquitously expressed transmembrane receptor found on endothelial cells of the lungs, vasculature, and heart. Acute COVID infection can trigger an inflammatory response through a cytokine storm, prompted by infected endothelial cells releasing many inflammatory markers [6]. This inflammatory response to COVID infection causes oxidative stress to the cardiovascular endothelium and may result in vasculitis, increased vascular permeability, fibrin deposition, vascular thrombosis, and subsequent embolization [7].

Cardiac Injury Following COVID Infection

Mechanisms for long-term cardiac damage following COVID infection are still not fully understood. However, it has been well-established that COVID can infect the angiotensin-converting enzyme 2 (ACE2) receptors in the heart [8]. Additionally, the increased release of inflammatory mediators in the heart may cause myocarditis through the infiltration of immune cells into the myocardial tissue and the release of reactive oxygen species [9]. Cardiac injury following infection is most common in patients with comorbidities that increase the risk of cardiovascular disease such as hypertension and diabetes mellitus [7]. Chronic myocardial fibrosis following a cardiac injury can also lead to long-term decreased ventricular compliance, myocardial perfusion, and contractility of the heart muscle, resulting in decreased cardiac output and subsequent exercise intolerance [8].

Decreased Oxygen Consumption Following COVID Infection

Exercise intolerance following COVID infection has been measured quantitatively and qualitatively by calculating maximal oxygen consumption (VO_2 max_) with six-minute walk tests and other exercise tests. Given the effects of COVID infection on the cardiovascular system, it is not surprising that studies have shown that VO_2_ is lower one to three months following illness in patients with COVID compared to control groups. Following hospital discharge, patients infected with COVID demonstrated a roughly 35% decrease in peak VO_2_ compared to a control group of patients [10]. Prolonged use of a ventilator during severe COVID illness can also lead to pulmonary injury and lung restriction, which can further decrease the ability of the lungs to effectively transport oxygen. In another literature review by Nazir A et. al., within the first year following hospitalization for COVID infection, approximately 43% of these patients had obstructive lung diseases and 53% of patients with COVID had restrictive lung diseases. These findings suggest that COVID may cause significant lung impairment and reduction in VO_2_ contributing to exercise intolerance through the impairment of gas exchange at the alveolar level [4].

Deconditioning and Musculoskeletal Disorders Following COVID

Fatigue is one of the most common symptoms of COVID infection, presenting during the acute phase of infection and potentially persisting for several months. The cytokine storm that is triggered by the acute phase of infection can lead to extended high levels of interleukins that promote systemic inflammation and chronic fatigue [11]. As a result, many patients require bed rest during recovery, which, when prolonged, can lead to muscle atrophy and deconditioning resulting in decreased exercise tolerance.

Myalgia and arthralgia are also common symptoms of acute COVID infection and PASC and are likely due to multiple factors. Patients with acute COVID infection may develop acute myopathy and mild to severe muscle weakness. Patients with severe COVID infection may be at higher risk for developing critical illness myopathy, which is another form of acute myopathy caused by infection [12]. Risk factors for developing myopathy following COVID infection include respiratory failure, need for ventilation and neuromuscular junction blocking agents, need for IV corticosteroid treatment, systemic inflammation, hypoxemia, and extended periods of inactivity or bed rest [13].

Several currently utilized treatment modalities for severe COVID infection may contribute to muscle wasting and deconditioning, slowing patients’ recovery. Patients with severe infections often require ventilation and the use of musculoskeletal junction blockers to immobilize the patient. If prolonged, this immobilization will lead to disuse atrophy and deconditioning [6]. IV corticosteroids are also a common treatment for severe COVID infection, which may promote a hypermetabolic state and cause muscle wasting [6]. Likely as a result of these factors combined with general bed rest, muscle atrophy has been observed as early as the first week of ICU admission in patients with severe COVID infection [4].

Febrile Illness and Exercise Intolerance

Fever is a common presenting symptom of COVID, with the febrile response aimed at assisting the immune system in fighting off the infection. While a febrile response can be beneficial in eliminating infection, fever can have multiple negative effects on exercise capacity. In the acute phase, febrile illness results in increased muscle catabolism and decreased nutrient accessibility for muscle, resulting in acute exercise intolerance [14,15]. During fever, autonomic stimulation raises the core body temperature and heart rate, resulting in an elevated metabolic rate [14]. Additionally, the body switches to lipolysis and proteolysis-based metabolism, resulting in cytokine-induced muscle catabolism. These physiologic responses help boost the immune system but decrease the accessibility of essential nutrients to the working muscle. Finally, during the febrile response, the production of antidiuretic hormone (ADH) is reduced to inhibit the sweat response and further raise the core body temperature, which impairs the body’s natural cooling mechanisms and increases the risk of developing dehydration [14]. During exercise in a healthy person, high levels of metabolism increase heat production, which is offset by sweating. However, in febrile patients, a naturally elevated body temperature combined with the inhibition of antidiuretic hormone (ADH) prevents proper cooling, resulting in dehydration, dramatically reduced cardiac output, and subsequent exercise intolerance. For these same reasons, participating in exercise during a febrile illness may actually worsen the illness and increase the risk for complications and mortality [15]. Following the resolution of the infection and fever, these effects will be reversed though it may take several weeks to months to fully recover.

Post-Infection Fatigue Syndrome (PIFS)

As discussed above, there is an acute decrease in strength and endurance immediately following a febrile illness. However, in some patients, symptoms may persist and result in chronic exercise intolerance and long-term fatigue, termed post-infection fatigue syndrome (PIFS). When symptoms of fatigue persist for six months or more following infection, a diagnosis of PIFS may be considered [16]. The mechanism behind PIFS is complex and not well understood, but some studies have shown evidence of decreased cerebral oxygen consumption and blood flow as well as a discrepancy between feelings of fatigue and lack of actual muscle weakness [17]. While muscle strength is maintained, muscle recovery has been shown to be impaired during PIFS. This may be due to a lack of blood flow, impairing the delivery of oxygen and removal of waste from the muscles, and thus delayed muscle recovery and low tissue oxygen saturation may be indicative of PIFS. It is important to note that PIFS occurs more frequently with specific infections, namely, infectious mononucleosis caused by the Epstein-Barr virus. PIFS as a component of long COVID, however, is a current topic of interest for researchers, as many patients are reported to experience PIFS for months following COVID infection [16].

Exercise Intolerance Due to Other Viruses

It is important to note that any symptomatic viral infection can cause exercise intolerance during the infectious period. However, there are multiple viruses that are exemplary of causing long-term post-infectious sequelae, which include Epstein-Barr virus (EBV), Human Immunodeficiency Virus (HIV), influenza, and Coxsackie virus [18-20]. Unfortunately, the pathophysiology of virus-induced exercise intolerance is not fully understood [21]. 

EBV is a common herpes virus that infects B cells and causes mononucleosis. While most cases of EBV will resolve without long-term sequelae, it is possible to develop chronic fatigue syndrome (CFS), characterized by persistent fatigue and sleep problems that can present as exercise intolerance. EBV may cause chronic immune system activation and subsequent inflammation resulting in exercise intolerance similar to other inflammatory diseases such as rheumatoid arthritis, lupus, and inflammatory bowel disease. Furthermore, it has been shown that EBV patients who develop CFS have reduced sensitivity to stress, thus suggesting that the hypothalamic-pituitary-adrenal (HPA) axis may be dysregulated [19]. Reduced sensitivity may lead to a blunted cortisol response and resultant abnormal cortisol levels. However, there is controversy over the cause of CFS. On the one hand, EBV may directly affect the HPA, which, in turn, may lead to a chronic inflammatory state but, conversely, a chronic inflammatory state may potentially instead cause HPA axis dysregulation.

HIV is mainly an infection of CD4-positive T cells and is also a cause of CFS. Classically, HIV is thought of as a deficiency in immune response, but recent studies have described the effect of HIV as dysregulation rather than simple deficiency due to the role CD4 T cells play in regulating the immune system’s pro- and anti-inflammatory components [22]. Prolonged infection with HIV may lead to a chronic inflammatory state, which has been theorized to result in exercise intolerance. Progressive HIV and eventual AIDS also lead to dramatic decreases in vitality due to severe infection, cancers, and autoimmunity. Because HIV can involve a multitude of systems, it is possible that the mechanism of HIV-induced exercise intolerance may vary from person to person. For example, two HIV patients, one with a lung tumor and one with pneumonia may both present with exercise intolerance.

Influenza is a common respiratory virus that can directly inflame and damage the respiratory tract and parenchyma, leading to CFS and exercise intolerance. This can decrease the oxygenating capacity and release cytokines that contribute to a chronic inflammatory state. Influenza may also affect skeletal and cardiac muscle and decrease strength [23]. Coxsackievirus has also been shown to directly involve muscle, but it seems to be more associated with skeletal muscle rather than cardiac muscle [20]. In summary, though the causal relationship between viruses and exercise intolerance has not been firmly established, future research on the effects of chronic inflammation and HPA axis regulation will be useful in studying the long-term effects of COVID and other viruses.

Severity of exercise intolerance in other conditions

Deconditioning Due to Bed Rest

As mentioned previously, prolonged bed rest can lead to muscle atrophy and deconditioning, resulting in exercise tolerance. Disuse presents a major risk factor for deconditioning in patients with severe COVID who require intensive care and mechanical ventilation. Physical inactivity alters muscle contractile properties leading to further physical inactivity and disuse. Studies have shown that muscle atrophy appears rapidly with a decrease in muscle mass of 5% in the first 10 days and up to 20% within the first six weeks [13]. In these studies, disuse-related loss of muscle accounted for ~80% of the decrease in muscle strength. Several studies have also shown a decrease in isometric muscle strength of 5-15% in patients with febrile illness requiring bed rest as compared to <4% in healthy control patients over the same period. Deconditioning not only reduces muscle strength but also reduces endurance capacity, with a 13-18% decrease in endurance capacity in those requiring bed rest due to febrile illness [15]. These results show that disuse can cause a loss of both strength and mass, resulting in lower force-generating capacity, increased fatigability, and subsequent exercise intolerance.

Acute Infectious/Febrile Illness

Deconditioning due to bed rest and exercise intolerance due to febrile illness are often related. As mentioned earlier, febrile illness leads to increased levels of pro-inflammatory cytokines, leading to generalized muscle catabolism and loss of muscle strength. Dehydration and body fluid regulation also contribute to exercise intolerance during febrile illness. Not only does muscle mass and muscle strength decrease but cardiac output and blood pressure also decrease significantly as well. Studies have shown stroke volume increases by 23-27% after the resolution of febrile illness [24]. A decrease in isotonic and isometric muscle strength of up to 30% as early as the first few days of febrile illness has also been demonstrated [25], as well as a strong correlation between reported myalgia and decreased muscle strength [15]. In summary, the effects of febrile illness are likely to extend beyond deconditioning and muscle loss with lasting negative effects on circulation and muscle metabolism.

Infectious Mononucleosis

Although EBV mononucleosis infection is associated with fatigue, few patients develop CFS. The prevalence of CFS six months after EBV infectious mononucleosis is thought to be between 9% and 12% [26]. This contrasts with COVID, which is commonly associated with fatigue and exercise intolerance, with up to 46% of patients reporting fatigue lasting weeks to months following COVID infection [16]. A meta-analysis of chronic fatigue in COVID patients four weeks after the onset of symptoms found the prevalence of chronic fatigue to be 45.2% among 127,117 patients [27]. However, a similar study published in JAMA, which studied 1.2 million individuals from 22 countries found the rate of fatigue with pain or mood swings to be a more modest 3.2% [28]. There appears to be a large variation between studies surveying long-term COVID fatigue.

Unfortunately, comparing the differences in severity between EBV and COVID presents a few challenges. Both diseases are multifactorial in nature, and disease severity is directly related to predisposing factors and comorbidities. Future studies may compare EBV and COVID using verified severity indices while controlling for comorbidities, but such a study has yet to be conducted. 

Severity of Exercise Intolerance Following COVID-19 Infection

Multiple studies have shown significant exercise intolerance over the course of COVID and following infection resolution. Roughly 80% of patients with moderate-to-severe disease demonstrated significant weakness at the time of discharge from the hospital [7]. Many tests, including sit-to-stand and short physical performance battery tests, measuring muscle strength and endurance averaged between 54% and 74% of predicted normal values [29]. de Andrade‐Junior et al. observed a ~30% reduction in rectus femoris cross‐sectional area in patients with severe COVID‐19 after ~10 days, which is a much greater loss than that seen in healthy individuals over the same time period [30]. Thirty days following hospitalization for COVID-19, 30%-50% of patients exhibit lasting exercise intolerance as noted by abnormal physical function or difficulty performing daily living activities [7]. When analyzing an average hospital stay, a significant delay in V_O2 max_ of 35% in COVID patients has been noted compared to the average hospital stay’s 4-6% reduction. V_O2 max_ was also found to be lower in patients infected with COVID 6 months post-hospitalization than other average hospitalized patients [10]. The severity of this exercise intolerance further suggests that moderate-to-severe COVID infection likely decreases exercise intolerance through multiple mechanisms including deconditioning, febrile illness, and damage to the respiratory system.

These studies have highlighted the importance of studying the effects of COVID-19 infection on exercise intolerance. Evidence of prolonged cardiovascular damage and adverse effects on skeletal muscle function has been well-documented for many types of febrile illness, and so it is critical to continue to document the long-term effects of COVID infection on exercise intolerance.

Limitations of this review

This study is not without limitations. The search criteria were intentionally broad to capture a wide range of studies in multiple disciplines to increase finding generalizability. The use of keyword searching can result in omitting potentially important papers, but this was mitigated through the addition of more specific terms such as “cardiac”, “endothelial”, or “pulmonary”. Additionally, our study included selected articles consisting of diverse research methodologies, including quantitative, qualitative, and mixed methods studies. Given the heterogeneity of the literature, a narrative synthesis approach was deemed necessary to integrate the data into a comprehensive analysis that would not be possible using other study designs. As such, data extraction relies heavily on the interpretation of the authors, which has the potential to introduce bias. To reduce potential bias, a dual review of the literature was completed by two independent reviewers (E.W. and B.H.), with discrepancies resolved by a third reviewer (J.H.). Additionally, the use of large databases, such as PubMed, allowed for sampling from a large pool of research, which may reduce the bias introduced in single articles or journals. Narrative approaches such as this allows for the synthesis of multidisciplinary literature into a common theme to improve understanding of the topic. Finally, the study of exercise intolerance is an emerging field as is research on mechanisms regarding COVID-19 infection and “Long COVID”. As further research continues to elucidate the details of how COVID and exercise intolerance are related, these findings may be subject to change.

## Conclusions

This review summarizes current literature surrounding exercise intolerance following COVID infection in terms of mechanism, risk factors, and comparisons to similar viral conditions. Febrile illness and deconditioning, combined with cardiac, pulmonary, and endothelial dysfunction in the acute and chronic phases, likely contribute to lasting exercise intolerance in patients. Risk factors, such as respiratory disease, systemic inflammation, and prolonged bed rest in COVID patients, predispose patients to more severe deconditioning, exacerbating subsequent exercise intolerance. Additionally, the exercise intolerance seen following COVID infection has numerous similarities to common viral illnesses such as influenza. Following infection, if fatigue and exercise intolerance persist for six months physicians should consider the diagnosis of PIFS. As the first step of rehabilitation management involves functional assessment, clinicians may use tests such as sit-to-stand and short physical performance battery tests to assess and follow the patient's physical recovery. Most importantly, the prevalence and severity of exercise intolerance following COVID highlight the need for effective physical rehabilitation to restore muscular and cardiovascular function. These findings underscore the importance of the long-term management of patients with COVID and the need for ongoing research to identify effective treatments for exercise intolerance in this population.
